# Cuprous oxide nanoparticles trigger reactive oxygen species-induced apoptosis through activation of erk-dependent autophagy in bladder cancer

**DOI:** 10.1038/s41419-020-2554-5

**Published:** 2020-05-14

**Authors:** Qiao Xiong, Anwei Liu, Qian Ren, Yongping Xue, Xiaowen Yu, Yidie Ying, Hongliang Gao, Haoyuan Tan, Zhensheng Zhang, Wei Li, Shuxiong Zeng, Chuanliang Xu

**Affiliations:** 1 Department of Urology, Changhai Hospital, Second Military Medical University, Shanghai, P. R. China; 2 Department of Geriatrics, Changhai Hospital, Second Military Medical University, Shanghai, P. R. China; 3 Company 6 regiment 2, College of Basic Medicine, Second Military Medical University, Shanghai, P. R. China; 4 Laboratory of Nano Biomedicine, Second Military Medical University, Shanghai, 200433 P. R. China

**Keywords:** Drug development, Bladder cancer

## Abstract

Cisplatin-based chemotherapy is the first-line treatment for patients with advanced bladder cancer. However, as more than 50% of patients are ineligible for cisplatin-based chemotherapy, there is an urgent need to develop new drugs. Cuprous oxide nanoparticles (CONPs), as a new nano-therapeutic agent, have been proved to be effective in many kinds of tumors. In the present study, CONPs showed dose-dependent and time-dependent inhibitory effects on various bladder cancer cell lines (T24, J82, 5637, and UMUC3) and weak inhibitory effects on non-cancerous epithelial cells (SVHUCs). We found that CONPs induced cell cycle arrest and apoptosis in bladder cancer cells. We further demonstrated that the potential mechanisms of CONP-induced cytotoxicity were apoptosis, which was triggered by reactive oxygen species through activation of ERK signaling pathway, and autophagy. Moreover, the cytotoxic effect of CONPs on bladder cancer was confirmed both in orthotopic xenografts and subcutaneous nude mouse models, indicating that CONPs could significantly suppress the growth of bladder cancer in vivo. In further drug combination experiments, we showed that CONPs had a synergistic drug–drug interaction with cisplatin and gemcitabine in vitro, both of which are commonly used chemotherapy agents for bladder cancer. We further proved that CONPs potentiated the antitumor activity of gemcitabine in vivo without exacerbating the adverse effects, suggesting that CONPs and gemcitabine can be used for combination intravesical chemotherapy. In conclusion, our preclinical data demonstrate that CONPs are a promising nanomedicine against bladder cancer and provide good insights into the application of CONPs and gemcitabine in combination for intravesical bladder cancer treatment.

## Introduction

Bladder cancer is one of the most commonly diagnosed cancers worldwide, with around 549,000 new cases diagnosed each year^1^. Approximately 75% of the newly diagnosed cases of bladder cancer are non-muscle invasive bladder cancer (NMIBC) cases, whereas the remaining 25% are muscle invasive bladder cancer or metastatic disease incidents^2^. Cisplatin-based combination chemotherapy remains the standard treatment for metastatic bladder cancer, with an overall survival of around 9–15 months^3^. Recently, immunotherapeutic antibodies targeting programmed cell death protein 1 and its ligand programmed death-ligand 1 have been approved as a first-line treatment in cisplatin-ineligible patients. Treatment using immunotherapeutic antibodies improved overall survival and quality of life when compared with chemotherapy^4,5^. Intravesical Bacillus–Calmette–Guerin (BCG) immunotherapy has been the standard of care for high-risk NMIBC patients since 1976^6^. In clinical practice and in settings where BCG immunotherapy and cystectomy were either refused by patients or were not an option, multiple alternative intravesical agents were used; however, these agents yielded modest responses and were not durable^7^.

With the rapid development of nanotechnology and nanoscience, a large number of research studies have focused on exploring the properties and functions of nanomaterials, and create therapies for myriad of diseases advancing medical science and disease treatment^8^. Nanoparticles (NPs) possess unique properties, such as higher tissue permeability, larger surface-to-volume ratio, targeted drug delivery, and relatively low cost^9^. Nanomedicines have been emerging as one of the new therapeutic options when conventional therapies, such as chemotherapy and radiation therapy, are deemed ineffective^9,10^. Silicon nanowire^11^, gold NPs^9^, chitosan NPs^12^, lipid NPs^13^ and cationic polymers^14^ are some of the recently designed nanomedicines for use in tumor therapies. Trace elements, such as copper, are key elements of complex enzymes responsible for the regulation of the antioxidant systems in both animals and plants^15,16^. Meanwhile, these trace elements can also be toxic to cells and other organisms. Shen Nong’s Herbal, the traditional Chinese medicine textbook, describes a medicine containing azurite ore, a copper mineral that has potential antitumor efficacy^17^. Cuprous oxide nanoparticles (CONPs), an azurite ore representative, are the first type of copper nanomedicine found to be stable in various solutions^18^. In our previous study, we demonstrated that CONPs exhibit certain in vivo effects in melanoma^19^, renal cancer^17^, cervical carcinoma^20^ and prostate cancer^21^. Moreover, CONPs selectively targeted tumor cells, showed little systemic toxicity, and were rapidly cleared from organs in vivo^19,22^. CONPs could target mitochondria, inducing the production of reactive oxygen species (ROS) and eventually leading to cell apoptosis and death^18,22^. Its differential cytotoxicity between tumor and normal cells, good tolerance, and bioavailability in vivo make CONPs an attractive potential novel agent in the intravesical treatment of patients with bladder cancer.

In the present study, we aimed to evaluate the inhibitory effects of CONPs on bladder cancer both in vitro and in vivo. We performed combination studies with standard chemotherapeutic agents used for treating bladder cancer and CONPs. We further explored the potential underlying mechanisms of action of CONPs on bladder cancer cells.

## Materials and methods

### Cell lines and reagents

The bladder cancer cell lines (J82, T24, 5637, UM-UC-3) and non-cancerous SVHUCs used in the present study were purchased from the Cell Bank of the Type Culture Collection of the Chinese Academy of Sciences (Shanghai, China). The cells were cultured with the recommended medium at 37 °C in 5% CO_2_ according to the suppliers’ instructions. All cell lines were supplemented with 1% penicillin/streptomycin (Gibco, NY, USA). All cell lines used in this study were within 40 passages and authenticated by short tandem repeat profiling. Mycoplasma contamination was tested using a Mycoplasma Detection Kit (B39032, Biotool, Switzerland) according to the manufacturer’s instructions. The most recent cell line authentication and mycoplasma contamination tests were performed in June 2016. Antibodies against cleaved caspase-3, Ki-67, Cyclin B1, LC3B, ATG5, and ATG7 were purchased from Abcam (Shanghai, China). Antibodies against Phospho-p44/42 MAPK (Erk1/2) (p-ERK), p44/42 MAPK (Erk1/2) (t-ERK), p-mTOR, and GAPDH were purchased from Cell Signaling Technology (Danvers, USA). The Cell Counting Kit-8 (CCK-8) assay was purchased from Dojindo Laboratories (KUMAMOTO, Japan). The Cell Cycle Staining Kit and Annexin V–fluorescein isothiocyanate apoptosis detection kit were purchased from Lianke Biotech (Hangzhou, China). The ROS detection assay kit was purchased from Beyotime Biotechnology (Shanghai, China). The in situ cell death detection kit was purchased from Roche (Mannheim, Germany). The IHC DAB Kit was purchased from Zhongshan Jinqiao Biotech (Beijing, China). Gemcitabine and cisplatin were purchased from Yuanye Biotech (Shanghai, China) and Sigma-Aldrich (Shanghai, China) respectively. CONPs were synthesized according to the procedures previously described^22^.

### In vitro cytotoxicity assay

The cells in the exponential phase of growth were inoculated in 96-well plates at a concentration of 4000 cells per well. Stock solutions of cisplatin and gemcitabine were prepared in phosphate-buffered saline (PBS), and CONPs were prepared in the corresponding medium. After a 12 h incubation, the growth medium was replaced with fresh medium containing various concentrations of drugs or 0.2% dimethyl sulfoxide (DMSO) (control group). Cell viability was measured after treatment for an additional 48 or 72 h using the CCK-8 kit, according to the manufacturer’s protocol. Absorbance was detected by using a microplate reader (Bio-Tek, Georgia, USA) at 450 nm. Dose–response curves were generated with GraphPad Prism 7 software (GraphPad Software, Inc. La Jolla, CA, USA), and absolute 50% inhibitory concentrations (IC_50_) were calculated according to the method described by Sebaugh et al^23^. Combination index (CI) values were calculated using data obtained from cell viability assays and the CompuSyn software developed by Chou et al^24^. A CI value > 1 indicated antagonism, CI = 1 was additive, and CI < 1 indicated synergy. The CIs of the two drugs were also displayed using the Combenefit software developed by Di Veroli et al^25^ and available freely online (https://sourceforge.net/projects/combenefit/).

### Xenograft models

UMUC3 cells were suspended in PBS at a concentration of 1 × 10^7^ cells/ml before injection. For generating the subcutaneous bladder cancer model, 6- to 8-week-old female nude mice were sedated with 5% chloral hydrate (Sigma-Aldrich). Around 100 μl of cell suspension was injected subcutaneously into the shaved right hind leg of mice. Treatment was initiated when the average tumor diameter reached 5–7 mm after injection. CONPs were dissolved in a 5% glucose solution using an ultrasonic mixer (Shanghai Bilon Instrument, Shanghai, China). The mice in the CONPs treatment group were intratumorally injected with CONPs at a dose of 16 mg/kg (~400 mg CONPs) twice per week. Gemcitabine was administered by intraperitoneal injection at a dose of 150 mg/kg once a week for 4 weeks, whereas the control mice were injected with the same volume of a 5% glucose solution. Six mice per group were euthanized at different time points, and the tumors were obtained and weighed.

Twelve 5-week-old female NOD/SCID mice (Shanghai Laboratory Animal Center, SLAC, Shanghai, China) were used to establish the orthotopic bladder cancer model. The mice were anesthetized by intraperitoneal injection of chloral hydrate. A 24-G needle was inserted into the urethra of mice, and 30 μl hydrochloric acid (0.1 N) was infused and retained in the bladder for ~15 s. Then, 30 μl sodium hydroxide (0.1 N) was infused and retained for 15 s. After acute bladder urothelial mucosa injury, the bladder was subsequently washed with PBS. UMUC3 cell suspension (100 μl) transfected with luciferase reporter gene was then injected at a concentration of 1 × 10^7^ cells/ml into the mice bladder using a 24-G needle, and the urethra was ligated with suture to retain the cells for around 3 h. Following the confirmation of orthotopic model establishment by imaging, CONPs were administered into the bladder via the urethra at a dose of 16 mg/kg per week; 5% glucose solution was injected into the control group. Whole-body imaging of mice was monitored weekly by live-animal bioluminescence optical imaging using a luciferase gene activated by d-luciferin (150 mg/kg) assay kit (Gold Biotechnology, St Louis, MO, USA) and an IVIS Lumina II imaging system (PerkinElmer, Hopkinton, MA, USA). The mice were sacrificed 6 weeks after implantation of tumor cells, and the bladder weights were monitored. The major organs and whole bladder were harvested and fixed with 10% formalin.

All animal experiments were performed in accordance with the Guide for the Care and Use of Laboratory Animals and were approved by the Bioethics Committee of the Second Military Medical University, and all experiments were performed following the relevant guidelines and regulations of the Second Military Medical University.

### Measurement of ROS

Intracellular ROS levels were determined using a ROS detection assay kit (Beyotime Biotechnology, Shanghai, China). T24 and 5637 cells were treated with CONPs at a concentration of 0.625, 1.25, or 2.5 mg/ml for 24 h. In inhibitory studies, the cells were incubated with CONPs alongside 5 mM of the ROS inhibitor *N*-acetylcysteine (NAC) (Beyotime Biotechnology) or 10 mM of 3-MA autophagy inhibitor (Sigma-Aldrich) for 2 h. The cells were then harvested, washed three times with PBS, and treated with 10 mM dihydrodichlorofluorescein diacetate (DCF-DA) in serum-free McCoy’s 5 A medium at 37 °C for 30 min. Then, the cells were washed 3 times with serum-free medium, after which DCF fluorescence was measured by using a MACS Quantify^TM^ flow cytometer (Miltenyi Biotec, Germany).

### Flow cytometry analysis

Apoptosis and necrosis were analyzed with the Annexin V–fluorescein isothiocyanate apoptosis detection kit (Lianke Biotech) following the manufacturer’s instructions. Cell cycle assay was assessed by flow cytometry using a cell cycle staining kit (Lianke Biotech) according to the manufacturer’s standard protocol. The samples were analyzed using a MACS Quantify^TM^ flow cytometer (Miltenyi Biotec), and the results were analyzed using the FlowJo v7.6.1 software (https://www.flowjo.com/).

### Western blot and immunohistochemistry (IHC) analyses

Western blot analysis and IHC staining were conducted according to the procedures previously described^26^.

### Terminal deoxynucleotidyl transferase (TdT)-mediated dUTP nick end labeling (TUNEL) assay

To test for apoptosis or necrosis in subcutaneous and orthotopic tumors, tissue blocks were sectioned into 3-mm slices and mounted on glass slides. The TUNEL assay was then performed using the in situ cell death detection kit (Roche), according to the manufacturer’s instructions.

### Statistical analysis

All experiments were repeated at least in triplicate. Data are presented as the mean values and standard deviations. Continuous parametric data were subjected to Student *t* test or one-way ANOVA analysis. Chi-square test was used to compare categorical data. Kaplan–Meier survival curve and log-rank test were used to compare the overall survival of mice. A *P* value of less than 0.05 was considered statistically significant. SPSS 19.0 (IBM Inc.) or GraphPad Prism 5 (GraphPad Software, Inc.) was used for statistical analysis.

## Results

### CONPs inhibit bladder cancer cell proliferation, migration, and invasion

**Fig. 1:**
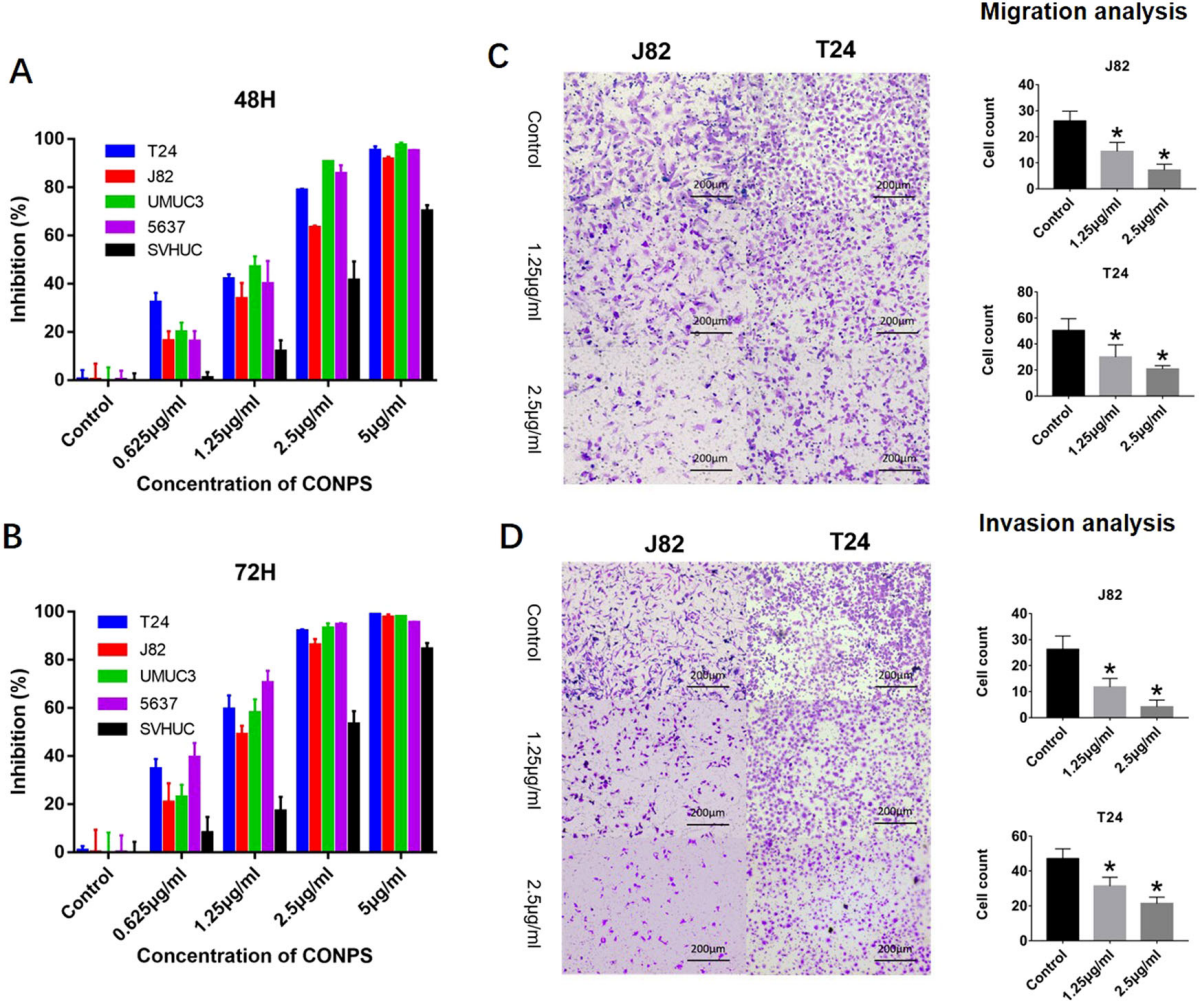
Effect of CONPs treatment on bladder cancer cell lines. **a**,**b** Differential cytotoxicity exhibited by CONPs on bladder cancer cell lines (T24, J82, UMUC3, 5637) and non-cancerous urothelial cells (SVHUCs) determined in a 48 and 72 h CCK-8 assay (*n* = 5). **c** CONPs significantly inhibited the migration capacity of J82 and T24 cells in the Transwell migration assay (*n* = 3). **d** Matrigel invasion assay demonstrated that CONPs significantly inhibited the invasion ability of J82 and T24 cells (*n* = 3). *P < 0.05.

CONPs exhibited cytotoxicity in bladder cancer cell lines in a dose- and time-dependent manner (Fig. 1a, b). The IC_50_ values for CONPs in T24, J82, UMUC3, and 5637 cells were 1.328, 1.807, 1.262, and 1.4 μg/ml after 48 h treatment, and decreased to 0.934, 1.158, 1.101, and 0.799 μg/ml after 72 h treatment, respectively. However, the IC_50_ values in SVHUCs were 3.247 and 2.552 μg/ml after 48 and 72 h treatment, respectively.

We further performed a Transwell assay to characterize how CONPs affected the migration and invasion ability of bladder cancer cells. The migration of T24 and J82 cells was significantly inhibited in a dose-dependent manner (Fig. 1c). The Matrigel invasion chamber assay also demonstrated that CONPs suppressed the invasion of T24 and J82 cells in a dose-dependent manner (Fig. 1d).

### CONPs induce cell cycle arrest and apoptosis

**Fig. 2:**
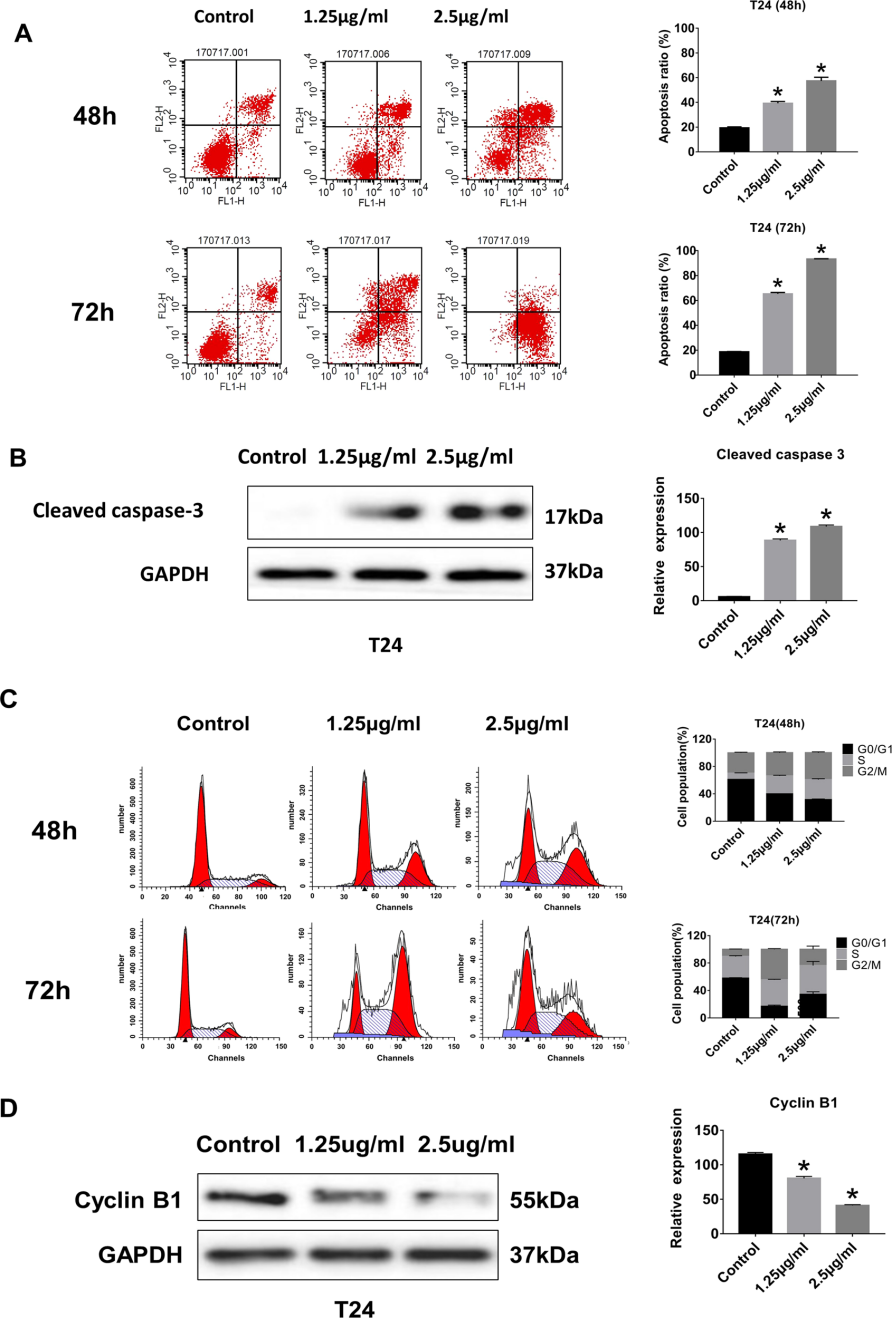
Treatment with CONPs induced apoptosis and cell cycle arrest in bladder cancer cells. **a** T24 cells were treated with different concentrations of CONPs at specified time points. The chart illustrates significant apoptosis induced by CONPs in a dose- and time-dependent manner. **b** The expression of cleaved caspase-3 was determined by western blot analysis after treatment of T24 cells with CONPs for 48 h. **c** T24 cells were treated with different concentrations of CONPs at specified time points, and cell cycle progression was arrested following CONPs treatment. **d** Western blot analysis showed reduced expression of the cell cycle checkpoint protein cyclin B1 after CONPs treatment for 48 h. *P < 0.05 (*n* = 3).

To elucidate the potential mechanisms underlying the cytotoxicity of CONPs in bladder cancer cells, we conducted flow cytometry to analyze the effects of CONPs administration on apoptosis and the potential disruption of cell cycle phases. Our results revealed a significant increase in the apoptosis of T24 and 5637 cells in a dose- and time-dependent manner (Fig. 2a and Supplementary Fig. S1). As shown in Fig. 2b, CONPs markedly increased the expression of cleaved caspase-3, suggesting that CONP administration activated the apoptosis signaling pathway in bladder cancer cells. Cell cycle analysis showed that treatment with CONPs significantly increased the percentage of G2/M phase cells compared to control cells in a dose-dependent manner (Fig. 2c and Supplementary Fig. S1). Further, western blot analysis demonstrated that CONPs could inhibit the expression of cyclin B1, a cell cycle regulatory protein predominantly expressed during G2/M phase of the cell cycle. These results indicated that the cytotoxicity exhibited by CONPs treatment in bladder cancer cells might be attributed to the activation of apoptosis and induction of cell cycle arrest at G2/M phase.

### CONPs suppress the growth of bladder cancer in tumor-bearing mice

**Fig. 3:**
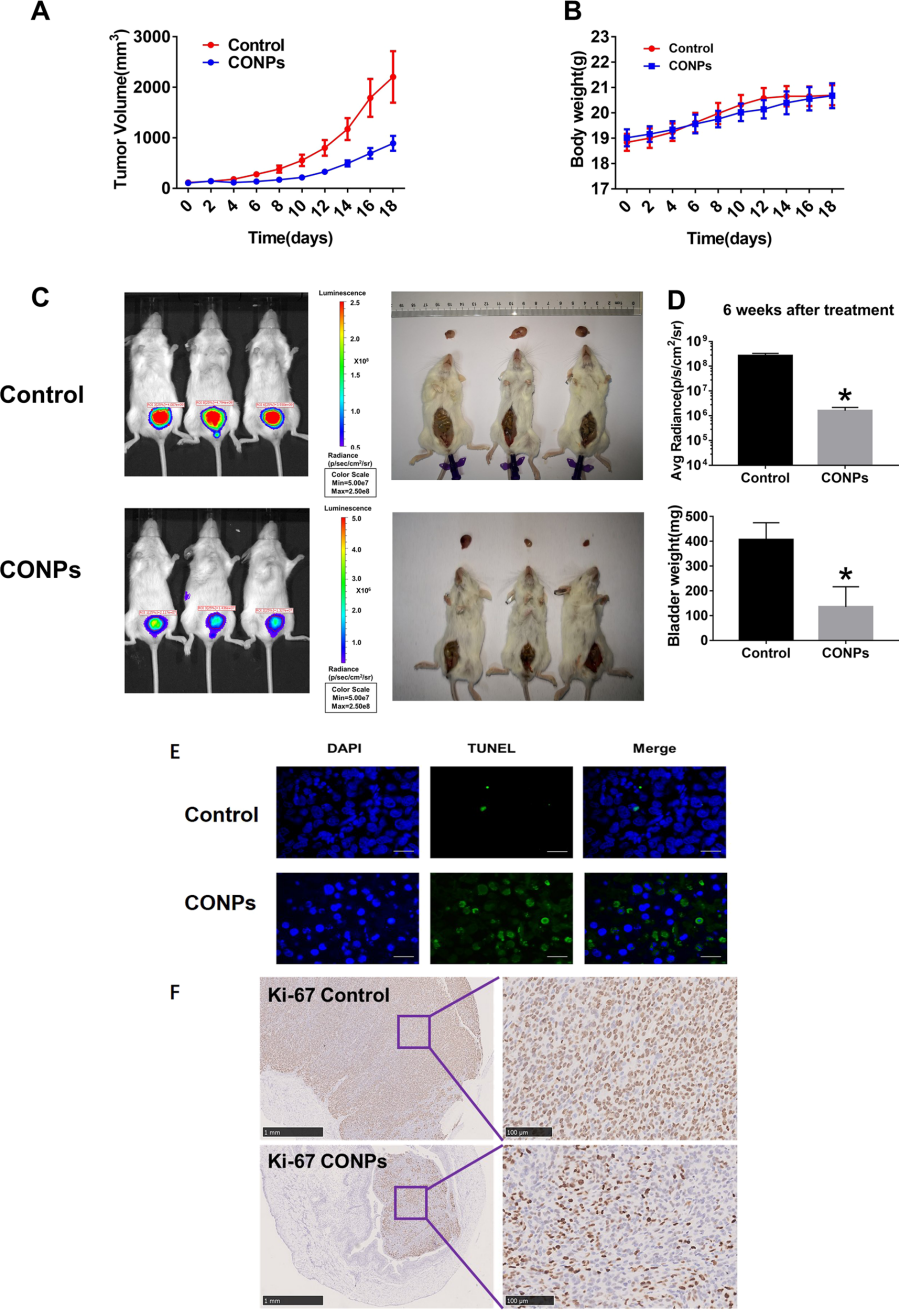
Antitumor efficacy of CONPs treatment on subcutaneous and orthotopic bladder cancer xenograft mouse models. **a** Tumor growth curve of subcutaneous nude mouse models established from T24 cells. Tumors grew more slowly in the CONPs treatment group: the median time to achieve a tumor volume of five times the baseline increased from 10 days in control to 15 days in the CONPs treatment group (*P* < 0.01, *n* = 6). **b** Body weights were measured every 2 days, and no obvious effects were observed on the body weight of mouse models following CONPs treatment (at day-14, *P* > 0.05). **c** Orthotopic xenografts derived from implantation of UMUC3 cells with stable luciferase expression were treated weekly with intravesical CONPs instillation, and tumor growth was monitored using an in vivo imaging system. **d** CONPs treatment group showed significantly lower radiance signals and bladder weight after 6 weeks than the control group. (*P* < 0.05, *n* = 6). **e** Representative images of TUNEL staining of dissected orthotopic tumors 3 days after intravesical instillation show that CONPs induced bladder cancer cell apoptosis in vivo. **f** Ki-67 expression in tumors obtained from orthotopic models markedly reduced in the CONPs group compared with the control group.

Next, we tested whether the cytotoxic effect shown by CONPs could be translated into in vivo antitumor activity in subcutaneous and orthotopic bladder cancer nude mouse models. Treatment with CONPs significantly inhibited tumor growth in the subcutaneous mouse model (Fig. 3a). The median time to achieve a tumor volume of 5 times the baseline increased from 10 days in control to 15 days in the CONPs treatment group (*P* < 0.01). No significant difference in body weight was observed in the CONPs treatment group when compared with the control (*P* > 0.05 at day = 14, Fig. 3b). In the orthotopic mouse model, tumor volume in the CONPs treatment group was clearly lower than that in the control group after four weekly intravesical instillation of CONPs (Fig. 3c,d). Histology examination of major organs after CONPs intravesical instillation revealed no obvious morphological changes in either the CONPs treatment group or control group (Supplementary Fig. S2). Immunohistochemistry and immunofluorescence staining of orthotopic tumors collected at day = 3 after CONPs intravesical instillation revealed that TUNEL-positive cells increased substantially (Fig. 3e and Supplementary Fig. S3), whereas Ki-67-positive cells reduced significantly (Fig. 3f) in the treatment groups.

### CONPs may induce apoptosis by activating ERK-dependent autophagy through triggering ROS

**Fig. 4:**
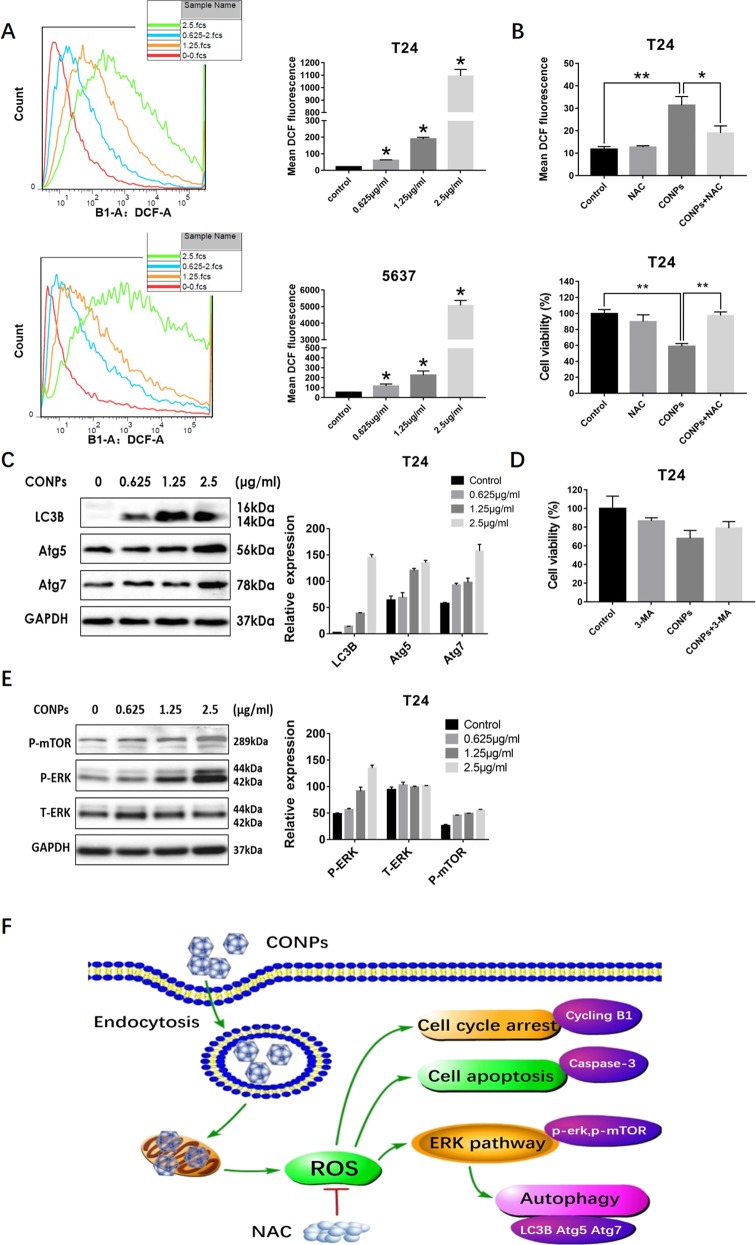
CONPs treatment induced autophagy via activation of the ROS/ERK signaling pathway in bladder cancer cells. **a** Bladder cancer cells (T24 and 5637) were treated with CONPs for 48 h and the ROS levels were measured by flow cytometry. CONPs treatment induced ROS generation in a dose-dependent manner. **b** T24 cells were preincubated with 5 mM of the ROS scavenger *N*-acetylcysteine (NAC) for 2 h before CONPs treatment. Our results suggested that ROS generation and CONPs-induced cell death were significantly inhibited and rescued by NAC administration, respectively. **c** The expression of LC3B, ATG5, and ATG7 was measured by western blot analysis 24 h after treatment with varying concentrations of CONPs. CONPs treatment led to the upregulated expression of these autophagy-related proteins. **d** Cell viability assays suggested that administration of the autophagy inhibitor 3-MA suppressed CONPs-induced cell death slightly. **e** Levels of phospho-ERK and total-ERK phospho-mTOR were determined by western blot analysis 24 h after treatment with varying concentrations of CONPs. The expression of phospho-ERK and phospho-mTOR proteins was upregulated by CONPs treatment in a dose-dependent manner. **f** Pathway diagram showing the potential molecular mechanisms through which CONPs can induce bladder cancer cell death. *P < 0.05 (*n* = 3).

In our previous study, we have demonstrated that CONPs could specifically target mitochondria, causing cytochrome C release, which in turn could further lead to ROS production^19,22^. ROS levels were determined by flow cytometry, which revealed that CONPs treatment could trigger ROS production in a dose-dependent manner (Fig. 4a). As displayed in Fig. 4b, NAC reduced CONPs-induced ROS production and rescued cell death as demonstrated by the cell viability assay. Meanwhile, autophagy is known as a double-sided cellular program that could both cause cell death and survival. In case of massive cell damages, autophagy could promote autophagic cell death^27^. We used western blot analysis to measure the expression of LC3B, ATG7, and ATG5. As shown in Fig. 4c, the expression levels of these proteins were consistently upregulated after treatment with CONPs in a dose-dependent manner, suggesting activation of autophagy. To clarify the interaction between autophagy and apoptosis, cell viability was measured in the presence of 3-MA. We observed a moderate decrease in dead cell numbers (Fig. 4d). The ERK pathway has been known to be activated and to induce the proliferation of malignant tumor cells^28^. However, there are reports indicating that ROS-dependent ERK activation could induce cell apoptosis and cell cycle arrest^29,30^. In addition, ERK signaling pathway was found to play an important role in autophagy^31^. In the present study, we revealed that p-ERK and p-mTOR protein expression was significantly enhanced by treatment with CONPs in a dose-dependent manner (Fig. 4e). Taken together, our results suggest that CONPs could activate the ERK signaling pathway by triggering ROS production, thereby further enhancing autophagic cell death (Fig. 4f).

### CONPs showed a synergistic drug–drug interaction with cisplatin and gemcitabine

**Fig. 5:**
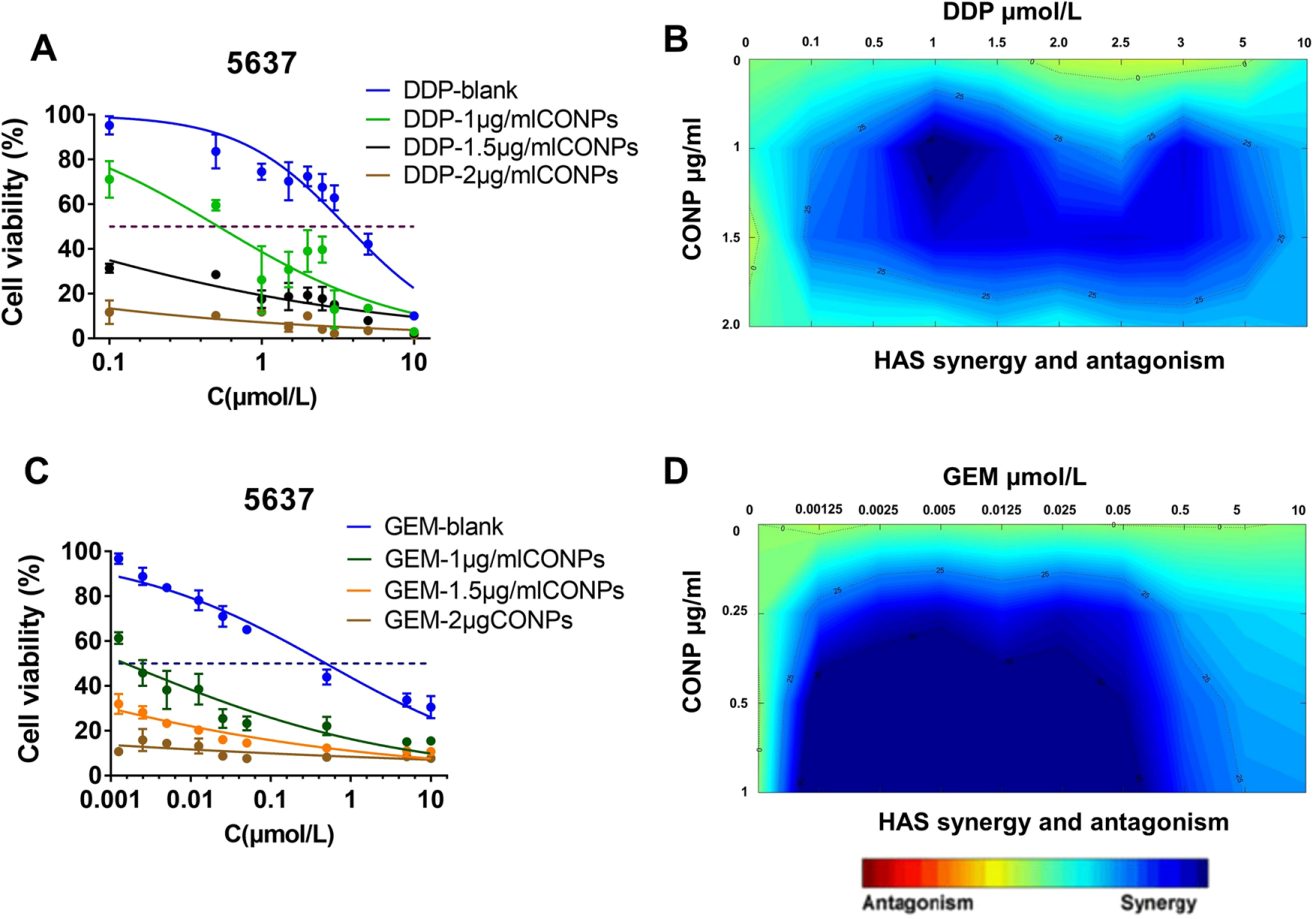
CONPs synergized with cisplatin (DDP) and gemcitabine (GEM) in vitro. **a** Dose–response curve of 5637 cells treated with varying concentrations of CONPs and DDP as determined in a 48 h cell viability assay. **b** Drug–drug interaction between CONPs and DDP analyzed using the Combenefit software based on the HAS model. Significant synergy is denoted by dark blue areas. **c** Dose–response curve of 5637 cells treated with varying concentrations of CONPs and GEM as determined in a 48 h cell viability assay. **d** Drug–drug interaction between CONPs and GEM. *P < 0.05 (*n* = 5).

Cisplatin-based combination chemotherapy has been the standard treatment for metastatic bladder cancer since 1980s^32^. Cisplatin combined with gemcitabine adjuvant chemotherapy is commonly used for patients with metastatic bladder cancer, prolonging survival for up to 14 months^33^. Meanwhile, gemcitabine could also be used for immediate intravesical instillation following transurethral resection of bladder tumor, significantly reducing the risk of recurrence^34,35^. We evaluated whether CONPs could potentiate the antitumor activity exhibited by cisplatin and gemcitabine. We used CI method to determine the drug–drug interaction between CONPs and cisplatin or CONPs and gemcitabine, respectively. The IC_50_ values of cisplatin gemcitabine were 3.628 and 0.4889 μmol/L for 5637 cells, and 0.7544 and 0.1315 μmol/L for T24 cells, respectively. Next, 5637 and T24 cell cultures were treated with increasing concentrations of cisplatin/gemcitabine in combination with CONPs. Compared with treatment with cisplatin or gemcitabine alone, CONPs enhanced the cytotoxicity displayed by cisplatin and gemcitabine in 5637 and T24 cells, which was indicated by a significant left shift of the dose–response curve in combination treatment with CONPs (Fig. 5a,c and Supplementary Fig. S4). The drug–drug interaction was analyzed with the Combenefit software^25^ using data from the cell viability assays. The contour plots of synergy/antagonism based on the HAS model are shown in Fig. 5b, d. These results indicated a synergistic effect between CONPs and gemcitabine or CONPs and cisplatin in 5637 and T24 cells (Supplementary Fig. S3). We further tested the CI values of these two combinations using CompuSyn^24^. These results also demonstrated that the combination of CONPs with gemcitabine or cisplatin in T24 and 5637 cells (Supplementary Fig. S3) showed synergistic effects.

### CONPs potentiated the antitumor activity of gemcitabine in vivo

**Fig. 6:**
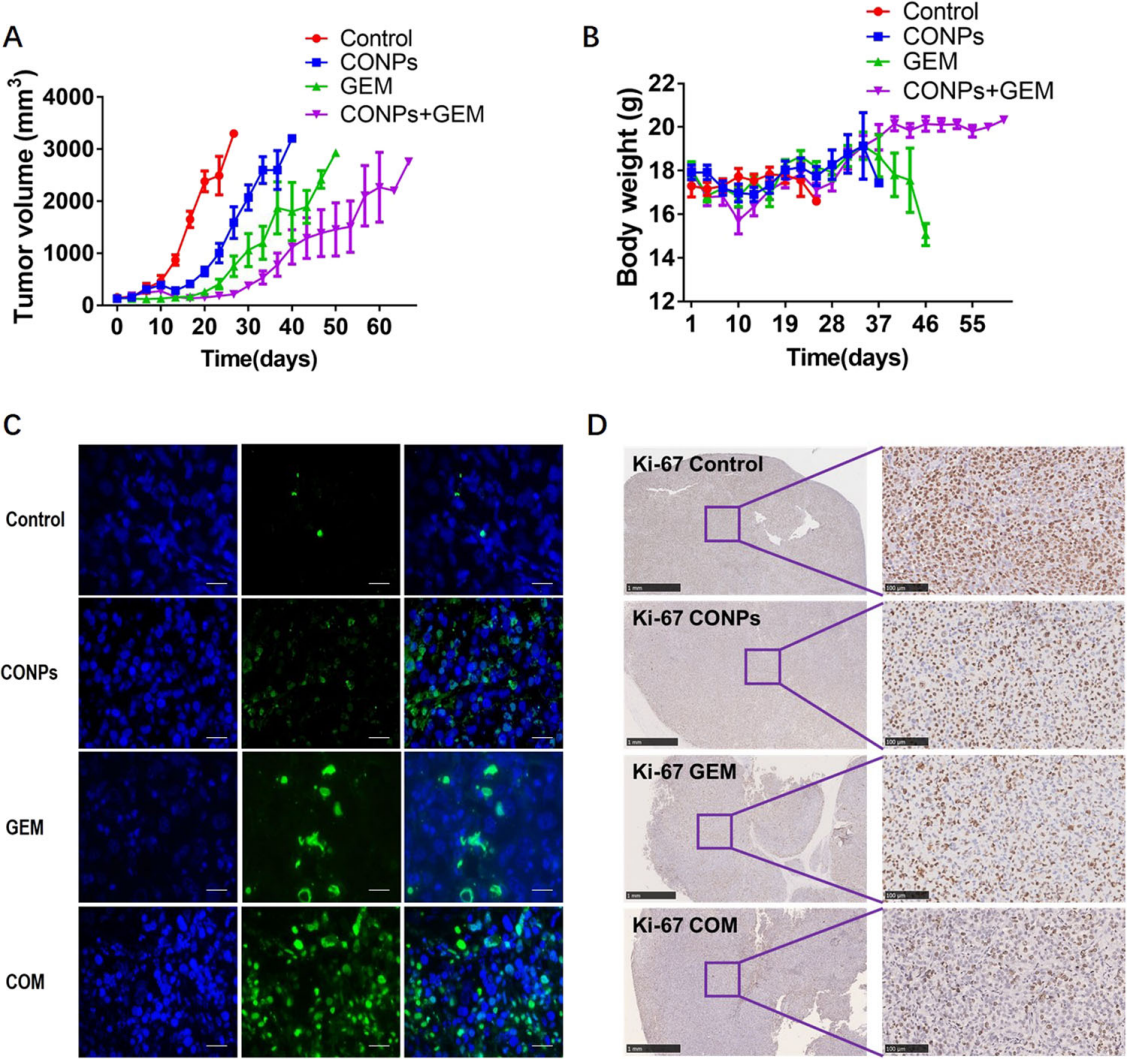
CONPs synergized with gemcitabine (GEM) in vivo. **a** Tumor growth curve of subcutaneous nude mouse models established by implantation of T24 cells. Tumors grew more slowly in all treatment groups relative to the control group. The median survival of control, CONPs, GEM, and combination treatment groups was 20, 34, 40, and 50 days (*P* < 0.05; control vs CONPs, *P* = 0.0017; CONPs+GEM vs GEM, *P* = 0.0264, *n* = 6), respectively. **b** No obvious effect on body weight was observed after either single drug or combination drug administration in the treatment groups at day-20 (*P* > 0.05). The body weights of mice in the GEM group gradually decreased owing to increased tumor burden, and they were promptly euthanized when losses in body weight reached > 20% of baseline. **c** Representative images of TUNEL staining of dissected subcutaneous tumors 3 days after drug treatment in each group revealed that the drug treatment groups induced more obvious apoptosis than the control group. **d** Representative images of Ki-67 expression 3 days after drug treatment. Ki-67 expression decreased markedly in all treatment groups compared with the control group.

We further determined whether the synergistic effect displayed by the combination treatment could be translated to in vivo antitumor activity. We selected CONPs and gemcitabine for the in vivo experiment because gemcitabine has been used for both systemic and intravesical therapy of bladder cancer. Combination intravesical therapies have only been recently undertaken^36^. Therefore, the synergistic effect exhibited by the combination of CONPs and gemcitabine might be closer to clinical practice. We compared the antitumor efficacy of single-agent CONPs and gemcitabine and combination treatment in nude mice subcutaneous bladder cancer tumors generated from implantation of T24 cells. We found that the in vivo results correlated well with the in vitro efficacy of CONPs and gemcitabine. As shown in Fig. 6a and Supplementary Fig. S5, the median time of tumor growth to 10 times the baseline size increased from 16 days for the control to 26 days for CONPs treatment, 33 days for gemcitabine treatment, and 42 days for the combination treatment group. The median survival of the combination treatment group (50 days) was significantly longer than that of the CONPs (34 days) or gemcitabine (40 days) treatment groups. Combination treatment significantly delayed tumor growth and prolonged lifespan when compared with single drug treatment with CONPs or gemcitabine (*P* < 0.01). Body weight decreased slightly in all treatment groups, while no significant difference was observed between single drug treatment groups and the combination treatment group (*P* < 0.05 at day-20, Fig. 6b). The number of TUNEL-positive cells was higher in the combination group than in single drug groups (Fig. 6c and Supplementary Fig. S6). In addition, immunohistochemical staining of tumors collected at 3 days after treatment initiation showed that the ratio of Ki-67-positive cells in the combination group was markedly lower than that in the single drug groups (Fig. 6d). These results suggested that combination treatment with CONPs and gemcitabine could potentiate their antitumor efficacy in vivo.

## Discussion

In this study, we comprehensively report both in vitro and in vivo antitumor ability of CONPs in bladder cancer. We found that CONPs inhibited proliferation of bladder cancer cells by inducing cell cycle arrest and apoptosis, and they displayed greater sensitivity against tumor cells than normal epithelial cells. As differential cytotoxicity is important in cancer therapy, this result suggested that CONPs might be a potentially useful intravesical anticancer drug. However, the difficulty of clearance of inorganic NPs from the body constitutes a bottleneck for their biomedical use^37^. For example, Ag NPs has been suggested to cause chromosomal imbalances^38^, and Au NPs was shown to disrupt zebrafish eye development^39^. To date, no gold-based nanomedicines have been approved, but rather only few iron oxide nanoparticles that have obtained US Food and Drug Administration approval^37^. Copper is essential to organisms as it serves as a co-factor for a variety of enzymes. Cuprous is the low valence state of the copper element, which may have even less toxicity^22^. We previously demonstrated that CONPs could be cleared from the tested organs of mice 7 days after a one-time injection; this could be a possible reason for the low toxicity exhibited by CONPs^19^. In the present study, we used CONPs to treat mouse subcutaneous and orthotopic bladder cancer models by intratumoral and intravesical injections, respectively. Our results suggested that CONPs significantly delayed tumor growth and prolonged the survival of bladder cancer xenograft models, with no obvious morphology damages in major organs. These results are consistent with our previous results, and further confirmed that CONPs could selectively induce apoptosis of bladder cancer cells.

Adjuvant intravesical therapy is routinely used after transurethral resection of NMIBC to decrease the odds of recurrence and progression^40^. For patients with high-risk NMIBC, maintenance BCG is the current recommended therapy^7^. However, BCG failure rate in the long term is as high as 50%, and a reliable alternative to radical cystectomy in BCG refractory disease remains controversial^41^. Currently, there is no standard intravesical therapy for NMIBC^42^. Recent studies suggested that administration of gemcitabine alone might be efficacious in cases of BCG failure, reaching recurrence free rates of 39–60%^42–44^. Combinations of multiple therapeutic approaches are an emerging trend toward optimizing the management of clinical diseases^45^. Tumor cells are a heterogeneous group characterized by varying genetic abnormalities, and therefore use of several drugs with different mechanisms of action, provide the possibility for maximal kill of tumor cells and reduces the chances of developing resistance to therapy^42^. Combination intravesical therapies have only been recently undertaken in the management of bladder cancer. Intravesical combinations, such as BCG and chemotherapy, doxorubicin and mitomycin C, gemcitabine and mitomycin C, as well as gemcitabine and docetaxel, have been reported in clinical trials with promising outcomes^36,46^.

To our knowledge, combined administration of nanodrugs plus chemotherapy as a potential intravesical therapeutic approach in bladder cancer has not been previously investigated. We found that CONPs combined with gemcitabine synergistically decreased the proliferation of bladder cancer cells both in vitro and in vivo. Gemcitabine is a pyrimidine analog that incorporates into actively replicating DNA, leading to the accumulation of strand breaks and ultimately to increased cell death^47^. In the present study, we revealed that CONPs could induce the apoptosis of bladder cancer cells by activating the ROS/ERK signaling pathway. Thus, CONPs and gemcitabine might kill bladder cancers cells more efficiently through their separate mechanisms of action than either drug alone, thereby reducing the chance of recurrence. Given the strong synergy and low systemic toxicity of CONPs and gemcitabine in bladder cancer, this combination treatment should be further validated in clinical trials.

Oxidative stress is an important phenomenon in cells, and plays a critical role in regulating both cellular survival and death in response to different stimuli, such as chemotherapeutic agents, starvation, and senescence^48,49^. We observed that CONPs could target mitochondria, resulting in the release of mitochondrial cytochrome C and activation of apoptosis pathways^19^. Mitochondria are an important site for ROS production^50^. Meanwhile, we found that CONPs could also increase ROS production by triggering endoplasmic reticulum stress and calcium release^18^. In the present study, we demonstrated that CONPs-triggered ROS production played a crucial role in inducing the death of bladder cancer cells, because the ROS scavenger NAC could significantly rescue cell death, whereas the autophagy inhibitor 3-MA could only demonstrate a modest rescue effect. We further explored the potential mechanisms of cell death resulting from enhanced ROS production and found that ROS induced the sustained activation of ERK signaling pathway. ROS can initiate and sustain ERK activation via different mechanisms, such as inhibition of tyrosine phosphatases and promotion of activation of tyrosine kinase receptors^29,51^. The ERK signaling pathway plays important roles in diverse aspects of cell functions including proliferation, differentiation, migration, and cell death^29^. Generally, the ERK signaling pathway is activated by various growth factors resulting in the proliferation of cancer cells^28^. However, ROS-dependent prolonged ERK activation could induce cancer cell cycle arrest and apoptosis, and it might be the crucial mechanism responsible for cancer cell death^29^. Similarly, other anticancer agents such as doxorubicin and cisplatin require prolonged ERK activation for inducing apoptosis in various cancer cells or immortalized cells^29,51^. This can explain the potential mechanisms underlying the synergistic effect between cisplatin and CONPs in the present study. As sustained ERK activity is required for promoting cell death, bladder cancer cells with activated ERK signaling might be more sensitive to CONPs than normal epithelial cells. Therefore, this mechanism might contribute to the selective toxicity exhibited by CONPs.

In conclusion, we demonstrated that CONPs are a promising nanomedicine against bladder cancer as they can selectively inhibit the growth of bladder cancer cells both in vitro and in vivo at a certain concentration. In addition, our results revealed that CONPs can lead to apoptosis and autophagy through the activation of ROS/ERK signaling pathway. Moreover, these preclinical data provide good insights into the application of CONPs and gemcitabine in combination for intravesical bladder cancer treatment.

## Supplementary information

Supplemental material
