# Supplementary material for: Cuprous oxide nanoparticles trigger reactive oxygen species-induced apoptosis through activation of erk-dependent autophagy in bladder cancer
Source: Cell Death Dis. 2020 May 14;11(5):366. doi: 10.1038/s41419-020-2554-5 (PMC7224387; doi:10.1038/s41419-020-2554-5)
Supplement: Supplementary file 1 — Supplemental material [file 41419_2020_2554_MOESM1_ESM.pptx]

## Slide 1
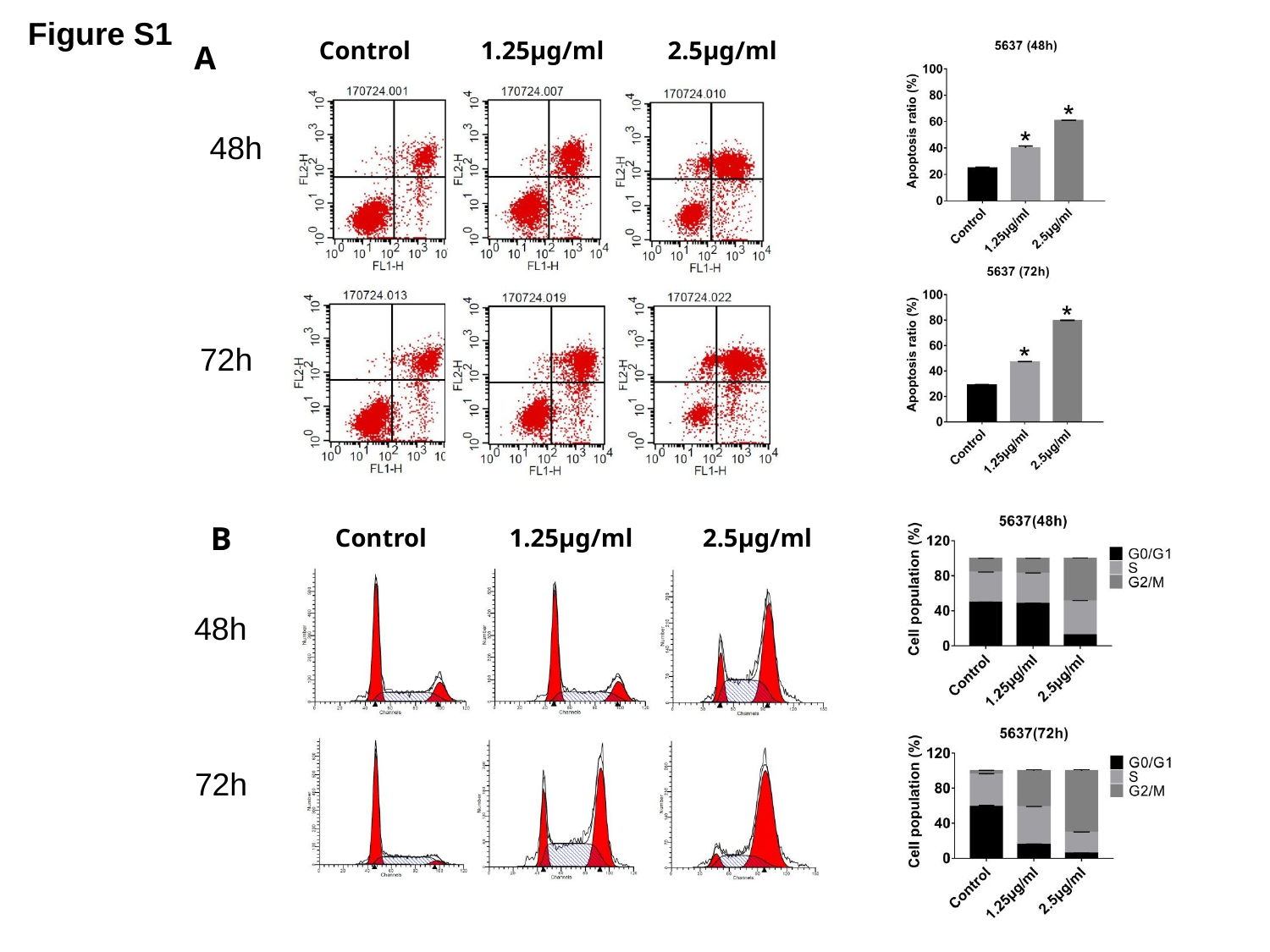

Figure S1
Control 1.25μg/ml 2.5μg/ml
48h
72h
B
Control 1.25μg/ml 2.5μg/ml
48h
72h
A

## Slide 2
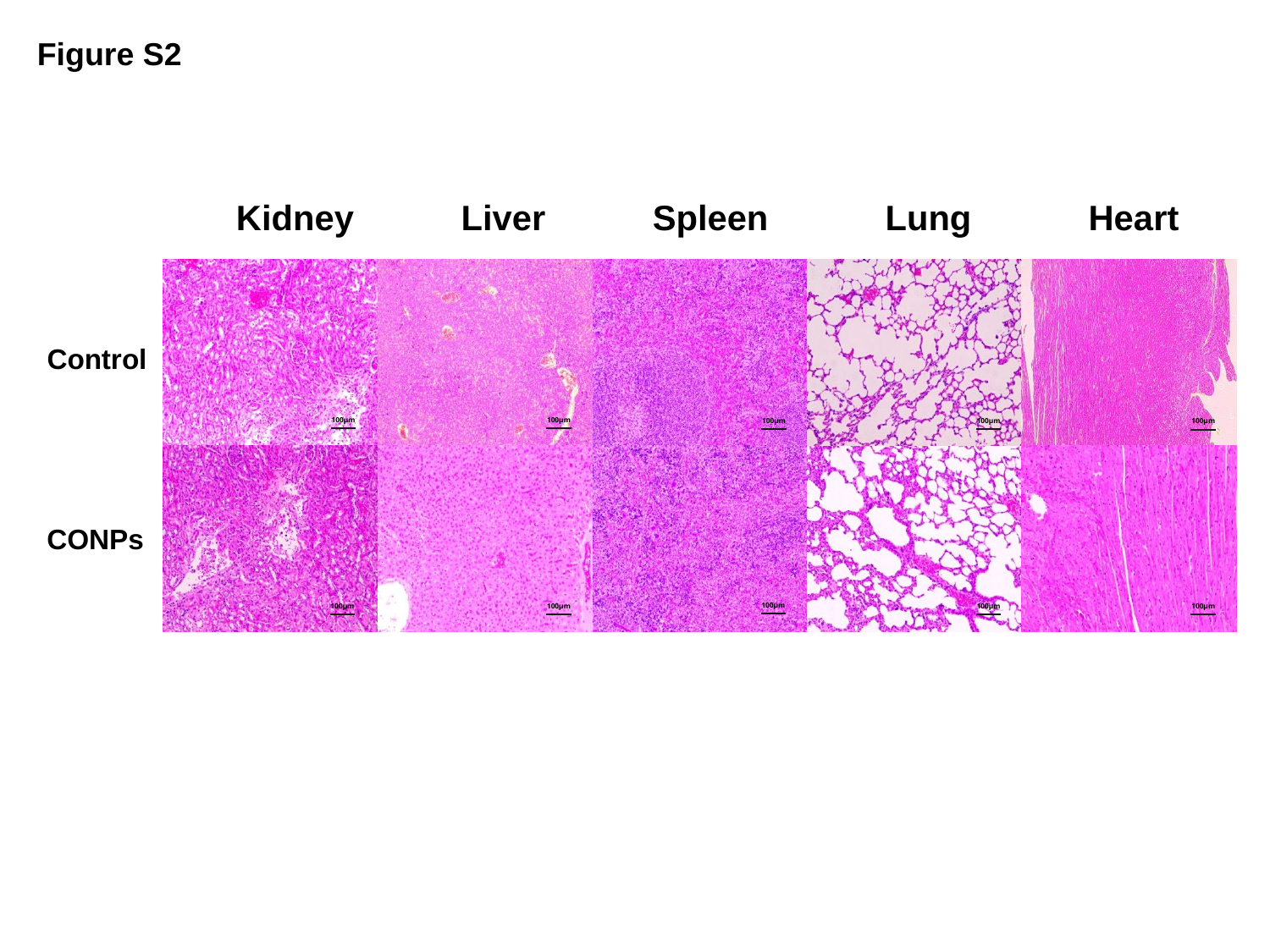

Figure S2
Kidney Liver Spleen Lung Heart
Control
CONPs
100μm
100μm
100μm
100μm
100μm
100μm
100μm
100μm
100μm
100μm

## Slide 3
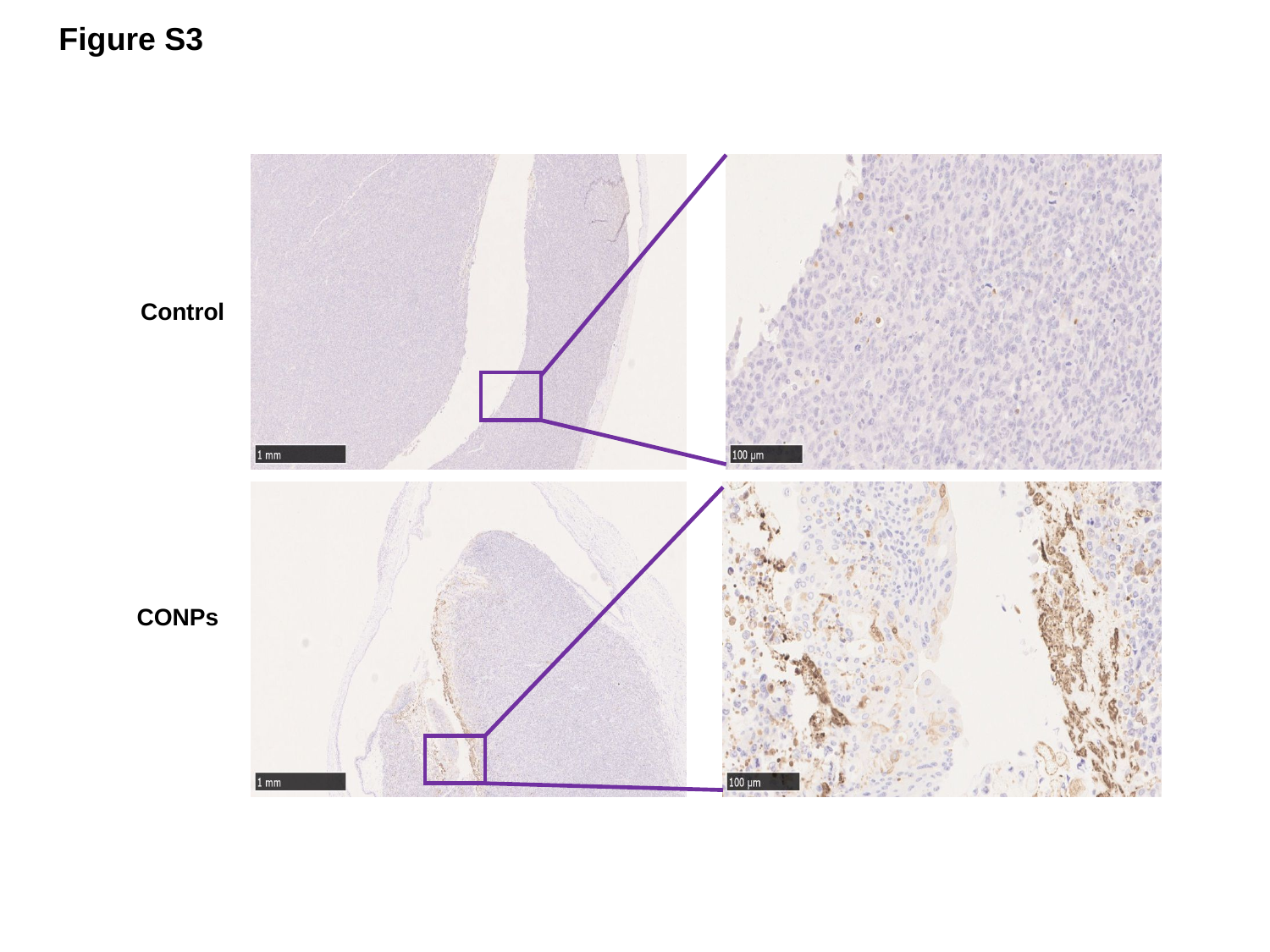

Figure S3
Control
CONPs

## Slide 4
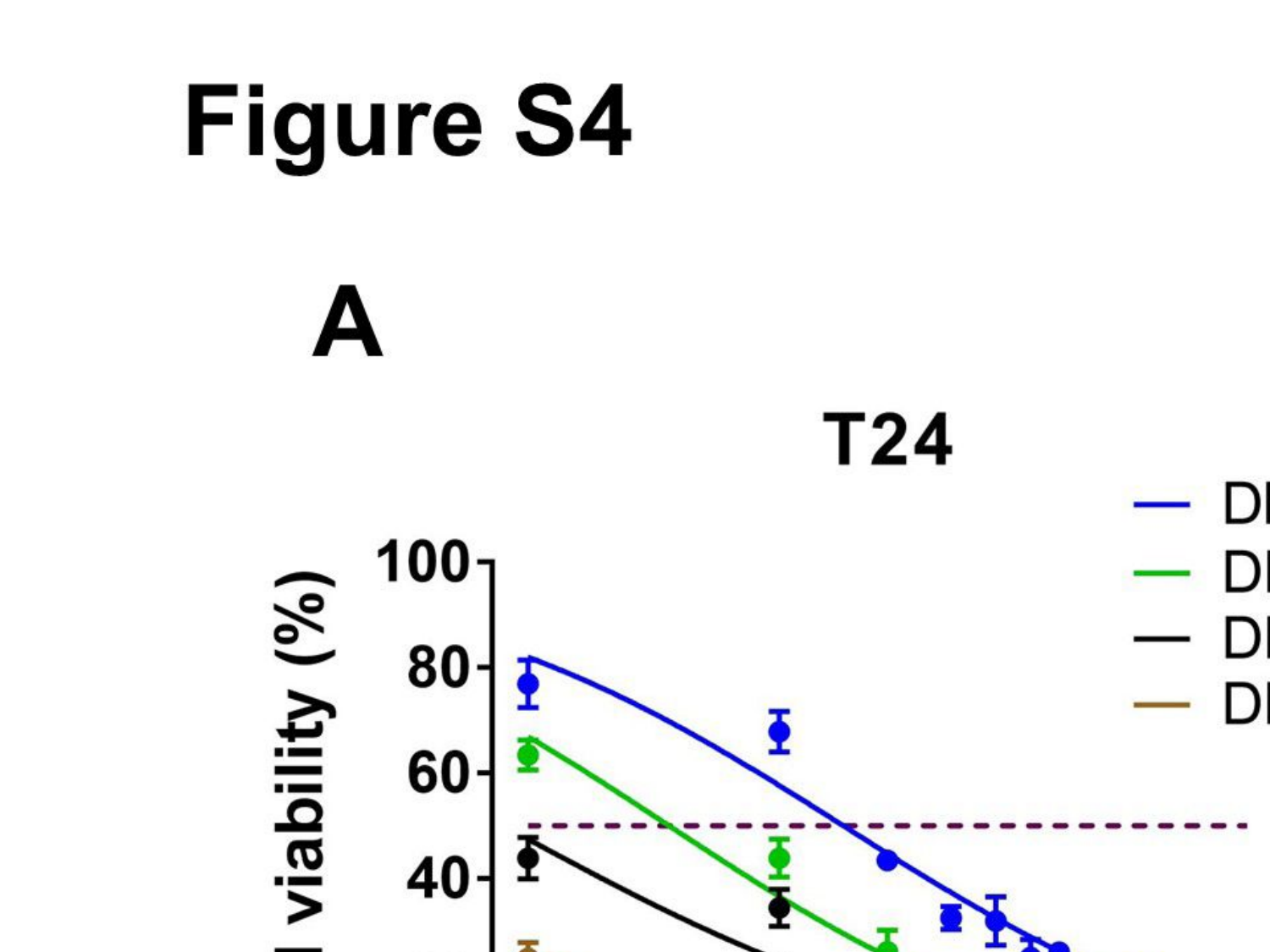

## Slide 5
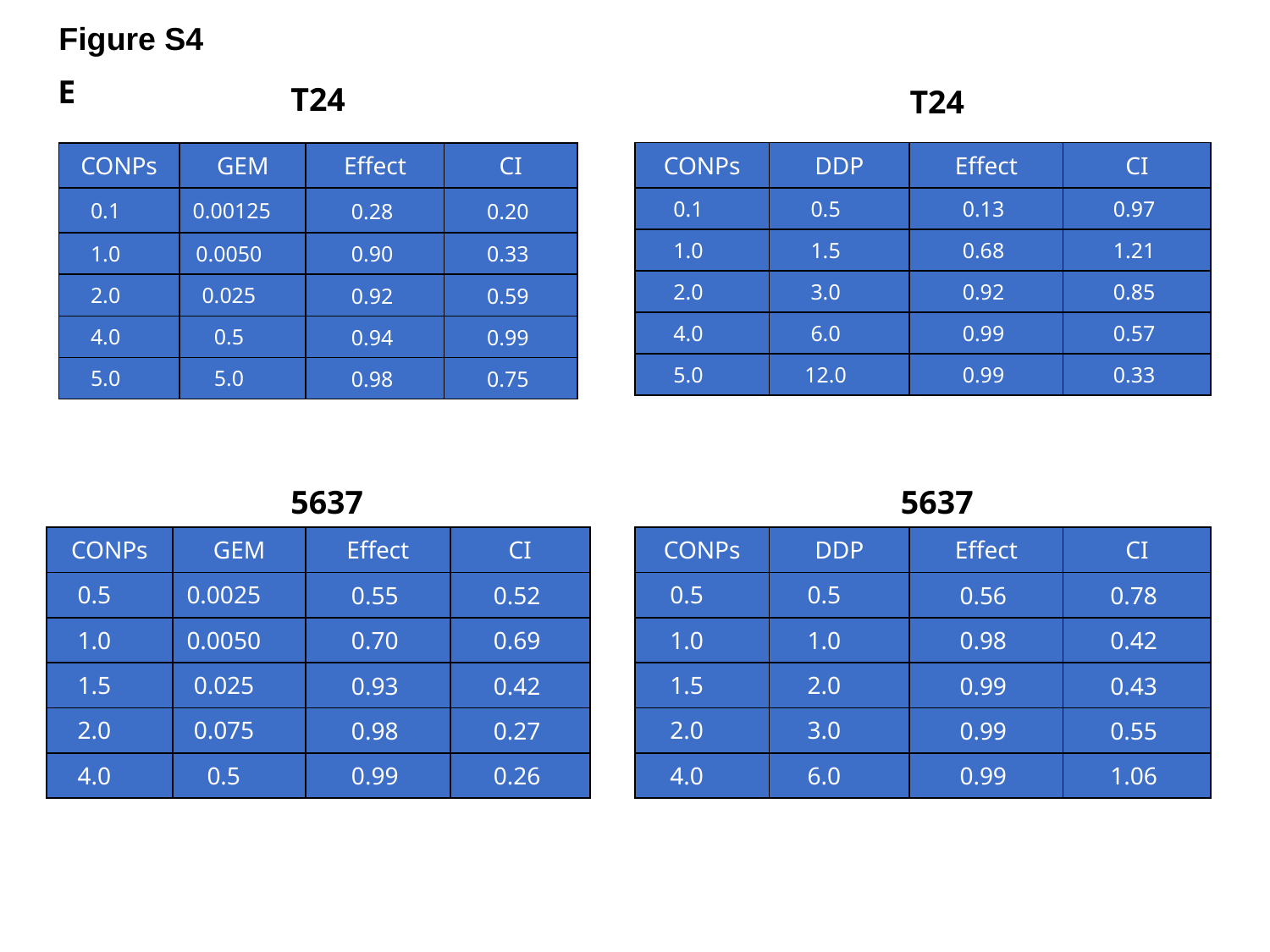

Figure S4
E
T24
T24
| CONPs | DDP | Effect | CI |
| --- | --- | --- | --- |
| 0.1 | 0.5 | 0.13 | 0.97 |
| 1.0 | 1.5 | 0.68 | 1.21 |
| 2.0 | 3.0 | 0.92 | 0.85 |
| 4.0 | 6.0 | 0.99 | 0.57 |
| 5.0 | 12.0 | 0.99 | 0.33 |
| CONPs | GEM | Effect | CI |
| --- | --- | --- | --- |
| 0.1 | 0.00125 | 0.28 | 0.20 |
| 1.0 | 0.0050 | 0.90 | 0.33 |
| 2.0 | 0.025 | 0.92 | 0.59 |
| 4.0 | 0.5 | 0.94 | 0.99 |
| 5.0 | 5.0 | 0.98 | 0.75 |
5637
5637
| CONPs | GEM | Effect | CI |
| --- | --- | --- | --- |
| 0.5 | 0.0025 | 0.55 | 0.52 |
| 1.0 | 0.0050 | 0.70 | 0.69 |
| 1.5 | 0.025 | 0.93 | 0.42 |
| 2.0 | 0.075 | 0.98 | 0.27 |
| 4.0 | 0.5 | 0.99 | 0.26 |
| CONPs | DDP | Effect | CI |
| --- | --- | --- | --- |
| 0.5 | 0.5 | 0.56 | 0.78 |
| 1.0 | 1.0 | 0.98 | 0.42 |
| 1.5 | 2.0 | 0.99 | 0.43 |
| 2.0 | 3.0 | 0.99 | 0.55 |
| 4.0 | 6.0 | 0.99 | 1.06 |

## Slide 6
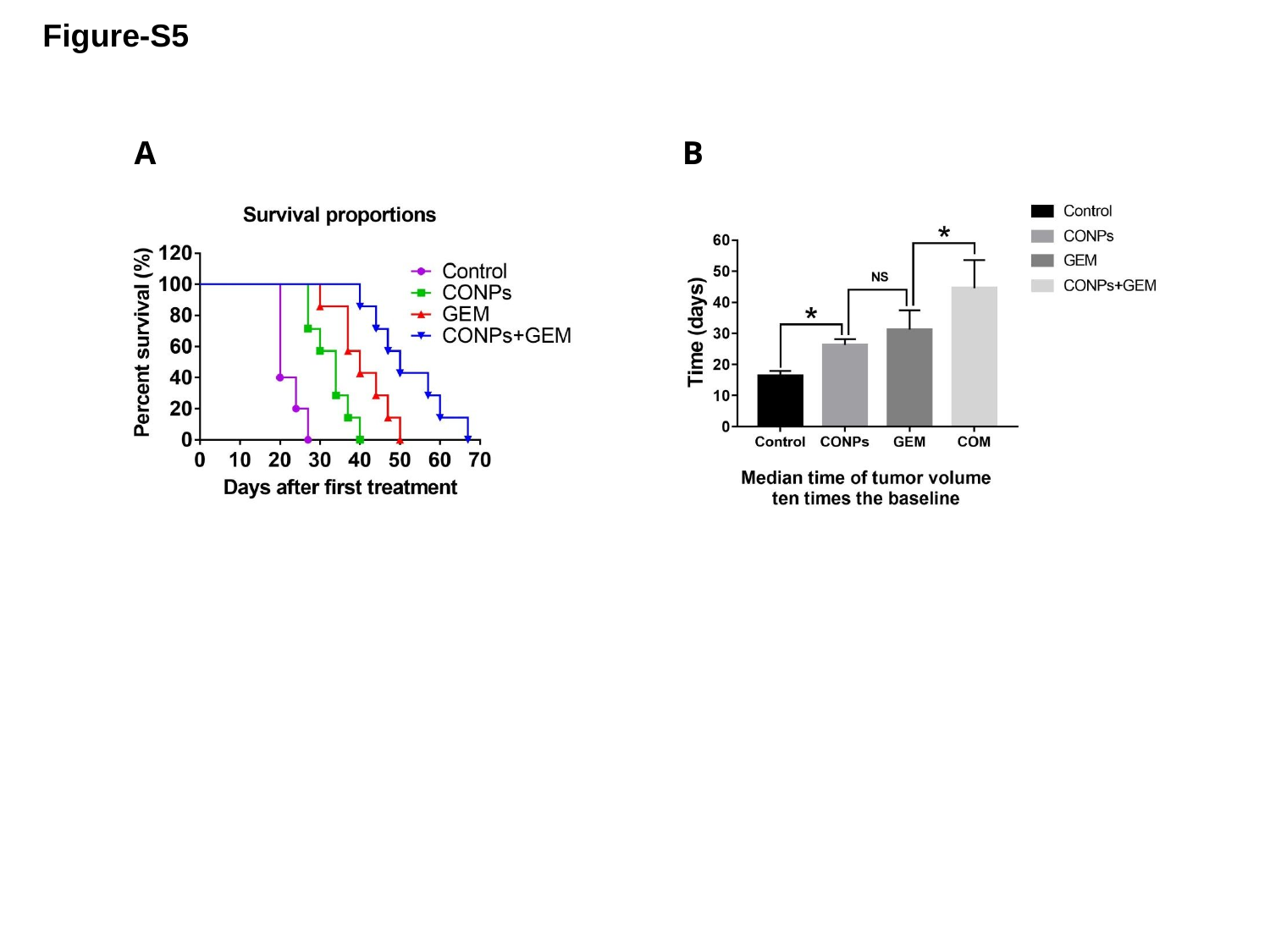

Figure-S5
A
B

## Slide 7
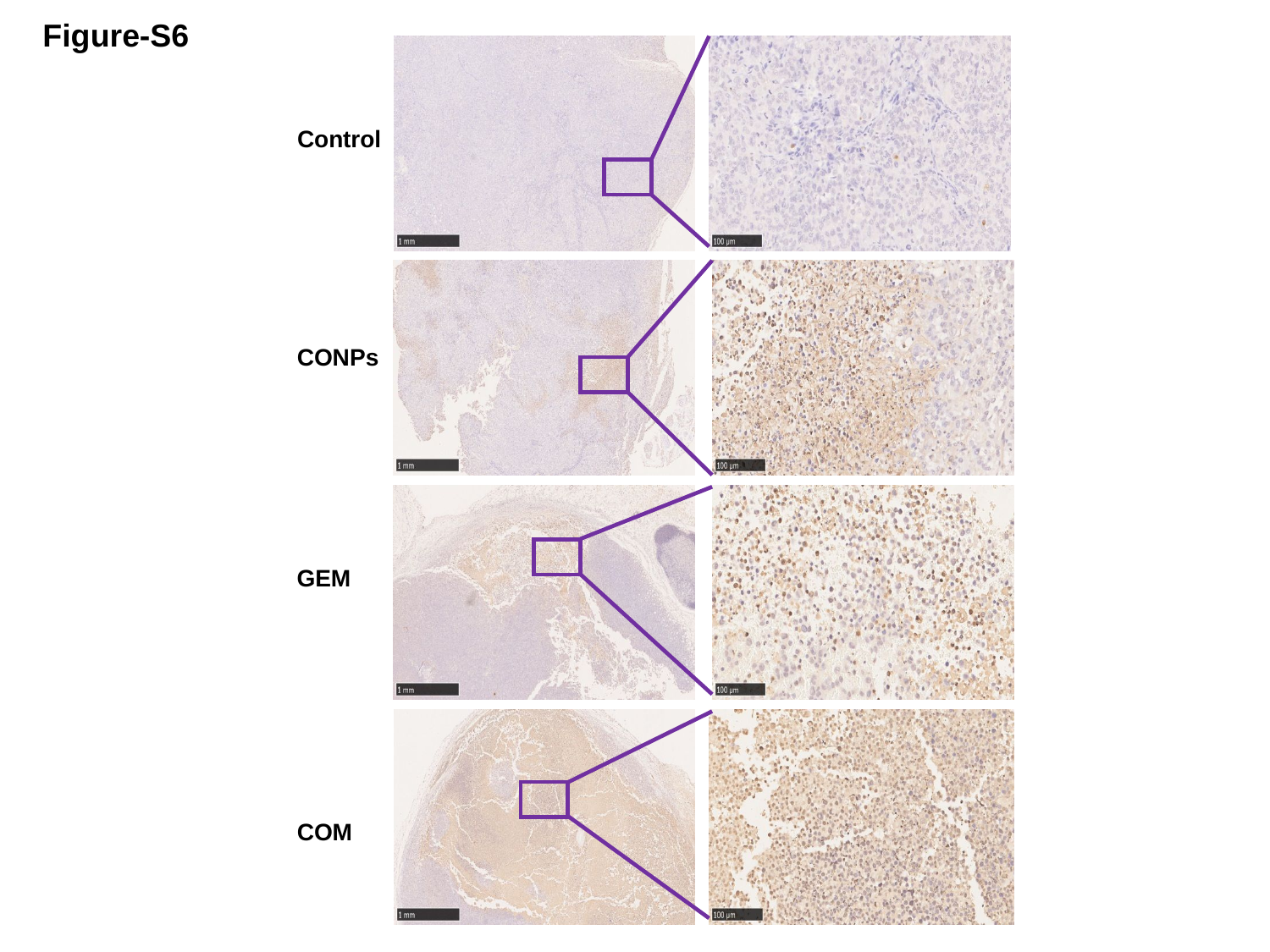

Figure-S6
Control
CONPs
GEM
COM
